# Soyasapogenols reduce cellular triglyceride levels in 3T3-L1 mouse adipocyte cells by accelerating triglyceride lipolysis

**DOI:** 10.1016/j.bbrep.2018.09.006

**Published:** 2018-09-27

**Authors:** Kazuko Iwamoto, Shuichi Kamo, Yuichi Takada, Ayana Ieda, Takatoshi Yamashita, Toshiro Sato, Nobuhiro Zaima, Tatsuya Moriyama

**Affiliations:** a School of Agriculture, Kindai University, Naka-machi, Nara, Nara 631-8505, Japan; b Product Development Laboratory, J-OIL MILLS, Inc., 7–41, Daikoku-cho, Tsurumi-ku, Yokohama-city, Kanagawa 230-0053, Japan; c Fundamental Research Laboratory, J-OIL MILLS, Inc., 7–41, Daikoku-cho, Tsurumi-ku, Yokohama-city, Kanagawa 230-0053, Japan; d KINDAI Research Institute for Agricultural Technology and Innovation, Naka-machi, Nara, Nara 631–8505, Japan

**Keywords:** Soyasapogenol A, Soyasapogenol B, 3T3-L1 adipocyte, Triglyceride, Lipolysis, Adipocytokine

## Abstract

Soyasapogenol is a soyasaponin aglycone, which has been suggested to exert a more potent function than the glycoside form. In this study, the effect of soyasapogenol A and B on cultured adipocyte cell function was investigated using mouse 3T3-L1 adipocyte cells. 3T3-L1 cells were treated with insulin, dexamethasone, and 3-isobutyl-1-methylxanthine for differentiation to adipocytes, and the cells were then cultured in the presence of soyasapogenol A or B (6.25 or 12.5 µM). The media were harvested and refreshed every 2 d. After a 10 d culture, the cells were harvested and the triglyceride content of the cells was determined. The triglyceride content of soyasapogenol B-treated cells was significantly lower than those of vehicle-treated cells. Glycerol and free fatty acid levels in the soyasapogenol-treated cell media were higher than those in vehicle cells. However, there was no difference in the level of adipose triglyceride lipase among soyasapogenol A-, soyasapogenol B-, and vehicle-treated cells. The secreted adiponectin and resistin levels of soyasapogenol-treated cell media were also different compared with those of vehicle-treated cells. Especially, the secreted resistin level in soyasapogenol B-treated cell media was obviously reduced compared with that of vehicle-treated cells. Taken together, these results suggest that soyasapogenol B exerted an anti-obesity and anti-diabetic effect on adipocytes by lowering the cellular triglyceride level by accelerating triglyceride lipolysis with reduced resistin secretion.

## Highlights

• Soyasapogenol A and B exerted an influence on TG accumulation in adipocytes.
• Soyasapogenol B accelerated lipolysis in adipocyte cells.
• Soyasapogenol-treated adipocytes significantly decreased resistin secretion.

## 1 Introduction

Metabolic syndrome (MS) is a group of metabolic factors that are associated with cardiovascular disease and type 2 diabetes [1], [2]. The risk factors of MS include abdominal obesity, visceral obesity, insulin resistance, dyslipidemia, high blood pressure, and glucose intolerance [3], [4]. Obesity is linked with the induction of insulin resistance in adipose tissue, which results in several pathologies related to type 2 diabetes. Studies that focus on food ingredients to improve obesity and lipid metabolism are expected to alleviate the pathological conditions of MS [5].

Soybeans have been reported to promote various health functions such as lipid metabolism [6]. They include many functional food components, such as soy protein, β-conglycinin, isoflavones, soy peptides [6], lectin, trypsin inhibitor, lecithin, tocopherol, and saponins. Their functional components have been reported to lower cholesterol and triglycerides and to improve lipid metabolism [7], [8], [9], although the exact mechanism of action exerted by those functional components has not been fully elucidated.

**Fig. 1:**
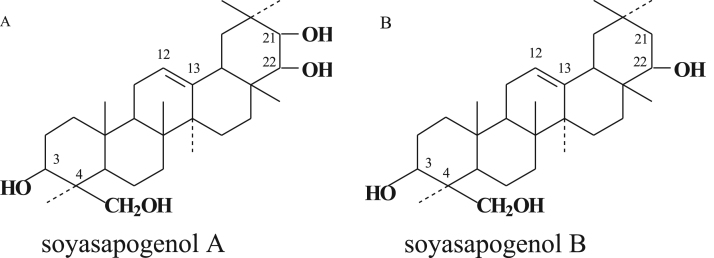
Chemical structure of soyasapogenol A and B. Chemical structure of soyasapogenol A (A) and soyasapogenol B (B).

Soyasaponins are triterpene glycosides that possess an oleanane-type aglycone with one or two polysaccharide chains [10]. Because of differences in aglycone compounds, soyasaponins are classified as group A, B, E, or 2,3-dihydro-2,5-dihydroxy-6-methyl-4H-pyran-4-one (DDMP) [11], [12]. As shown in Fig. 1, the structure of soyasapogenol A and B is very similar, but they differ in their number of hydroxyl groups. Group A soyasaponins, which have soyasapogenol A as aglycone, exhibit a more undesirable bitter taste than group B soyasaponins, which have soyasapogenol B as an aglycone [13], [14]. Soyasaponins have been reported to exert several functions, such as antioxidative [15], cholesterol-lowing [16], anti-kidney disease progression [17], anti-inflammatory [18], renin-inhibiting [19], hepatoprotective [20], antitumor [21], and anti-obesity [22]. In our previous report, the bioavailability of soyasapogenol (aglycone type) was shown to be better than that of a corresponding soyasaponin (glycoside type), and that of group B soyasaponins was shown to be better than that of group A soyasaponins [23], which suggested that soyasapogenols would be expected to have more health-promoting effects including lipid metabolism. However, a comparison of the anti-obesity effect between soyasapogenol A and B under an *in vitro* condition has not yet been reported. The purpose of the present study was to compare the *in vitro* biological effects between soyasapogenol A and B on cultured adipose cells using mouse 3T3-L1 cells.

## 2 Materials and methods

### 2.1 Sample preparation

Soyasapogenol A and B were purchased in powder form from Nacalai Tesque (Kyoto, Japan). They were first diluted in dimethyl sulfoxide (DMSO) and then added to a culture media for final concentrations of 6.25 and 12.5 µM. The final DMSO concentration was adjusted to 0.1% in the media.

### 2.2 Cell culture and adipocyte differentiation

Mouse 3T3-L1 (pre)adipocyte cells were purchased from the American Type Culture Collection (Manassas, VA, USA). They were maintained in Dulbecco's modified Eagle medium (DMEM) (Nissui Pharmaceutical Co., Tokyo, Japan) supplemented with 10% fetal bovine serum (FBS) (Gibco™, Life Technologies, Rockville, MD, USA), a 1% penicillin–streptomycin mixed solution, 200 mM L glutamic acid, 100 mM pyrubic acid, 4.5 g/L glucose, and 7.5% sodium carbonate at 37 °C under an atmosphere of 5% CO_2_ and seeded on 24-well plates. The day after the cells reached confluence (<90%), their differentiation into adipocytes was induced by using the Adipo Inducer Reagent (for animal cells, TAKARA BIO INC., Shiga, Japan) that contains insulin, dexamethasone, and 3-isobutyl-1-methylxanthine.

### 2.3 Sample treatment for adipocyte cells

Differentiated adipocytes were treated with DMEM supplemented with 10% FBS and soyasapogenol A and B (6.25 and 12.5 µM). The cells were then cultured and differentiated for 10 d, and the medium was refreshed every 2 d. Control cells were treated with 0.1% DMSO (final concentrations in the media) as vehicles.

### 2.4 Cytotoxicity assay

The cytotoxicity of soyasapogenol A and B was measured using the CellTiter96 Aqueous One™ Solution Cell Proliferation Assay kit (Promega Corporation, WI, USA).

### 2.5 Oil-red O staining

Intracellular lipid accumulation was detected using oil-red O staining (Nacalai Tesque). The adipocytes were rinsed with phosphate-buffered saline (PBS) twice and fixed in 10% buffered formalin for 10 min at room temperature. After rinsing with PBS twice, the cells were incubated with 60% isopropanol for 1 min. The staining started with oil-red O solution (oil-red O-saturated solution in isopropanol: water at 3:2) for 20 min. The cells were then washed with 60% isopropanol to remove background staining and finally rinsed with PBS. The stained cells were observed by microscopy.

### 2.6 Determination of triglyceride contents in cells

Mouse 3T3-L1 adipocytes were extracted by a radioimmunoprecipitation assay buffer (Nacalai Tesque) for the preparation of whole-cell proteins. Protein concentrations were determined by the Pierce™ BCA Protein Assay Kit (Thermo Fisher Scientific Inc., IL, USA). Measurement of the triglyceride level in 3T3-L1 adipocyte extracts was carried out by an enzymatic assay kits (TG Test Wako; Wako Pure Chemical Industries, Ltd., Osaka, Japan).

### 2.7 Determination of glycerol and free fatty acid concentrations in media

Glycerol levels in media were measured using the Glycerol Colorimetric Assay Kit (Cayman Chemical, MI, USA). Non-esterified free fatty acid (NEFA) levels in media were measured using the LabAssay™ NEFA (Wako Pure Chemical Industries, Ltd.).

### 2.8 Adipocytokine assay

Adiponectin and resistin levels in media secreted from cells were measured using enzyme-linked immunosorbent assay (ELISA) kits (Duo set, R&D Systems™, MN, USA).

### 2.9 Sodium dodecyl sulfate polyacrylamide gel electrophoresis (SDS-PAGE) and Western blot analysis

Thirty micrograms of protein from each lysate was boiled for 5 min in SDS sample buffer [24]. Proteins were separated by SDS-PAGE and transferred onto polyvinylidene difluoride membranes (Immobilon™-P, MerckMillipore, Billerica, MA, USA). Antibodies against adipose triglyceride lipase (ATGL) (MAB11192, R&D Systems™, MN, USA), peroxisome proliferator activated receptor γ (PPAR γ; H-100, Santa Cruz Biotechnology, Inc., TX, USA), fatty acid synthase (FAS; F9554, Merck, Darmstadt, Germany), β-actin (MAB1501, MerckMillipore), HRP-labeled anti-mouse IgG (Thermo Fisher Scientific Inc.), and HRP-labeled anti-rabbit IgG (Thermo Fisher Scientific Inc.) were also used for Western blotting. All immunoreacted proteins were detected using ECL™ Western Blotting Detection Reagents (GE Healthcare, Buckinghamshire, UK).

### 2.10 Statistical analysis

The statistical difference was determined by the Tukey–Kramer test. Differences were considered significant at *p* < 0.05. Statistical analyses were performed using Stat View v. 5.0 (SAS Institute, Tokyo, Japan).

## 3 Results

### 3.1 Effect of soyasapogenol A and B on cell viability and lipid accumulation in 3T3-L1 adipocytes

**Fig. 2:**
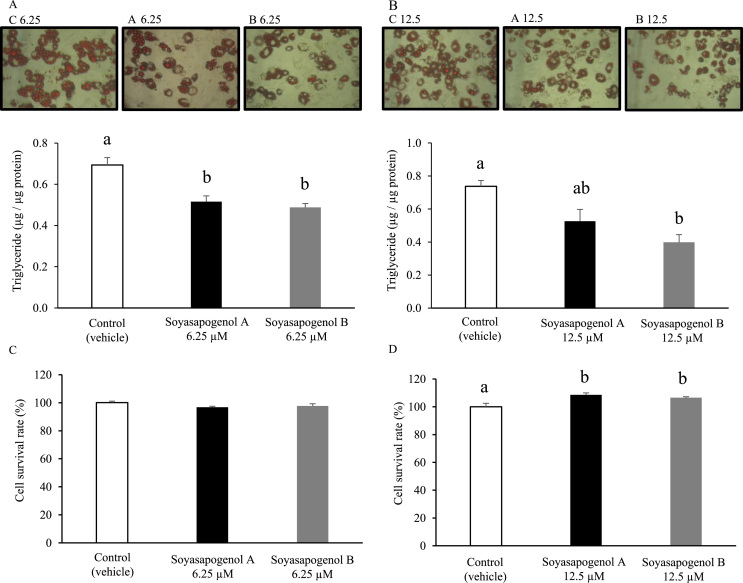
Effect of soyasapogenol A and B on lipid accumulation for 10 d in 3T3-L1 adipocytes. (A) Oil-red O staining and triglyceride content in 3T3-L1 cells treated with 6.25 µM soyasapogenol A or B; (B) oil-red O staining and triglyceride content in 3T3-L1 cells treated with 12.5 µM soyasapogenol A or B; (C) cytotoxic effect on the viability of 3T3-L1 cells treated with 6.25 µM soyasapogenol A or B; (D) cytotoxic effect on the viability of 3T3-L1 cells treated with 12.5 µM soyasapogenol A or B. Values are expressed as mean ± SE (*n* = 3). Values that do not share the same letter are significantly different (*p* < 0.05).

To determine the effect of soyasapogenol A and B on lipid accumulation in adipocytes, differentiated 3T3-L1 cells were exposed to soyasapogenol A or B for 10 d. The 3T3-L1 cells were stained by oil-red O staining to confirm the accumulation of lipid droplets (Fig. 2A and B). Although 6.25 µM soyasapogenol A reduced the triglyceride content in 3T3-L1 cells by 74.4%, 12.5 µM soyasapogenol A significantly reduced the triglyceride content by 71.4% compared with the vehicle. Also, 6.25 and 12.5 µM soyasapogenol B significantly reduced the triglyceride content in 3T3-L1 cells by 70.3% and 54.1%, respectively, compared with the vehicle.

There was no significant difference in triglyceride level between cells treated with soyasapogenol A or soyasapogenol B at either concentration. 3T3-L1 cell viability was measured using a cell proliferation assay kit. As shown in Fig. 2C, 6.25 µM soyasapogenol A and B did not alter 3T3-L1 cell viability (97% and 98%, respectively) compared with the vehicle. In Fig. 2D, 12.5 µM soyasapogenol A and B did not alter 3T3-L1 cell viability (109% and 106%, respectively) compared with the vehicle. These results indicate that up to 12.5 µM soyasapogenol A and B did not influence cell viability.

### 3.2 Effect of soyasapogenol A and B on lipolytic activity in 3T3-L1 adipocytes

**Fig. 3:**
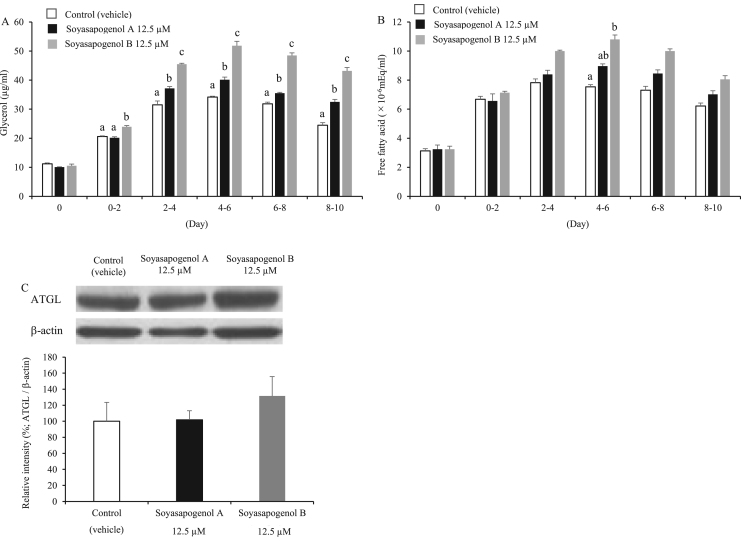
Effect of soyasapogenol A and B on lipolysis for 10 d in 3T3-L1 adipocytes. (A) Glyceride levels in a medium of 3T3-L1 cells treated with 12.5 µM soyasapogenol A or B; (B) free fatty acid levels in 3T3-L1 cells treated with 12.5 µM soyasapogenol A or B; (C) Western blot analysis showing ATGL activity in 3T3-L1 cells treated with 12.5 µM soyasapogenol A or B. Values are expressed as mean ± SE (*n* = 3). Values that do not share the same letter are significantly different (*p* < 0.05).

We further investigated whether soyasapogenol A and B inhibited lipid accumulation in 3T3-L1 adipocytes by analyzing glycerol and free fatty acid levels in media. As shown in Fig. 3A, the glycerol level in adipocyte cells cultured with soyasapogenol B was significantly higher compared with that in adipocyte cells cultured with either the vehicle or soyasapogenol A throughout the test period. In adipocyte cells cultured with soyasapogenol A, the glycerol level was significantly higher compared with that in the vehicle during the test period, besides days 0–2. No significant differences in free fatty acid levels were observed between adipocyte cells cultured with soyasapogenol A and those cultured with soyasapogenol B (Fig. 3B). These results suggest that soyasapogenol A and B enhanced lipolysis in adipocyte cells and accompanied the reduction of triglycerides in lipid droplets stained by oil-red O and that the potency of accelerating lipolysis was higher with soyasapogenol B than it was with soyasapogenol A.

### 3.3 Effect of soyasapogenol A and B on cellular ATGL protein levels in 3T3-L1 adipocytes

ATGL is one of the major regulators of lipolysis in adipocytes [25]. Therefore, we investigated the effects of soyasapogenol A and B on ATGL expression in 3T3-L1 adipocytes. As shown in Fig. 3C, ATGL expressions were not altered among the three groups.

### 3.4 Effect of soyasapogenol A and B on cellular lipogenesis levels in 3T3-L1 adipocytes

**Fig. 4:**
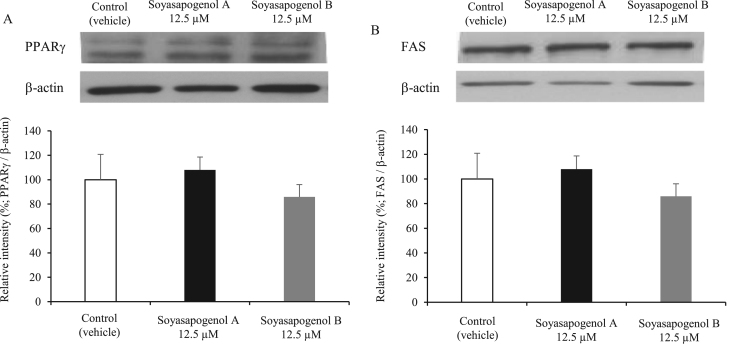
Effect of soyasapogenol A and B on lipogenesis for 10 d in 3T3-L1 adipocytes. (A) Western blot analysis showing PPARγ activity in 3T3-L1 cells treated with 12.5 µM soyasapogenol A or B; (B) Western blot analysis showing FAS activity in 3T3-L1 cells treated with 12.5 µM soyasapogenol A or B. Values are expressed as mean ± SE (*n* = 3).

To investigate the effect of soyasapogenol A and B on the expression of lipogenesis in 3T3-L1 cells, PPARγ and FAS levels were evaluated using western blot analysis. The expression of both the proteins was not altered among the three groups (Fig. 4A and B).

### 3.5 Effect of soyasapogenol A and B on adiponectin and resistin secretion from 3T3-L1 adipocytes

**Fig. 5:**
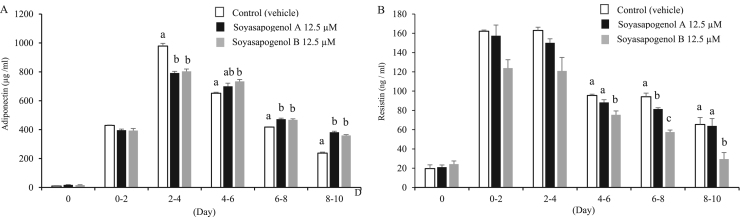
Effect of soyasapogenol A and B on adipocytokine secretion for 10 d in 3T3-L1 adipocytes. (A) Adiponectin concentration in a medium of 3T3-L1 cells treated with 12.5 µM soyasapogenol A or B; (B) resistin concentration in a medium of 3T3-L1 cells treated with 12.5 µM soyasapogenol A or B. Values are expressed as mean ± SE (*n* = 3). Values that do not share the same letter are significantly different (*p* < 0.05).

To assess the effect of soyasapogenol A and B on adipocytokine secretion from 3T3-L1 cells, adiponectin and resistin secretions from adipocyte cells were evaluated by quantitative sandwich ELISA kits. Although the secretion of adiponectin was significantly higher in adipocytes treated with soyasapogenol A and B compared with the vehicle from day 6–8 onward (Fig. 5A), the secretion of resistin was significantly decreased in adipocytes treated with soyasapogenol B compared with the vehicle from day 4–6 onward (Fig. 5B).

## 4 Discussion

Previously, Yang et al. [26] reported that soyasaponins Aa and Ab exert an anti-obesity effect in 3T3-L1 adipocytes through the downregulation of PPARγ. However, it was previously revealed that the bioavailability of soyasapogenol is better than that of corresponding soyasaponins and that the bioavailability of soyasapogenol B is better than that of soyasapogenol A [23]. Therefore, in the present study, we investigated the anti-obesity action of soyasapogenols, not soyasaponins, in adipocytes using 3T3-L1 cells.

First, we determined cell viability under two different soyasapogenol A and B concentrations; there were no toxicity findings in 3T3-L1 cells (Fig. 2C and D). We investigated the effect of soyasapogenol A and B on triglyceride content in adipocytes every 2 d after 10 d of culture. As shown in Fig. 2A and B, the oil-red O-stained intracellular triglyceride accumulation in soyasapogenols-treated cells was significantly reduced or was reduced compared with that in vehicle-treated cells. These results suggest that soyasapogenol A and B exerted an influence on triglyceride accumulation in adipocytes.

Intracellular triglycerides are generally hydrolyzed by ATGL and hormone-sensitive lipase to generate glycerol and NEFAs [25]. Lipolytic activity could be monitored by measuring the secreted glycerol and NEFAs in the media.

Glycerol and NEFA concentrations in media cultured with soyasapogenol B were significantly higher than in those cultured with the vehicle during the test period, suggesting that soyasapogenol B accelerated lipolysis in adipocyte cells accompanied with the reduction of triglycerides stored in lipid droplets. As the lipolysis of triglyceride in adipocytes is catalyzed by ATGL at a rate-limiting step [25], we studied the effect of soyasapogenol A and B on the protein expression of ATGL in the harvested cells after a 10 d culture. As shown in Fig. 3C, there were no differences in ATGL protein levels among the three groups. Therefore, the enhancement of lipolytic activity by adding soyasapogenol to the media might not be involved in changes in ATGL expression. The detailed mechanism of accelerating lipolysis by soyasapogenol is still unclear and should be resolved in the future.

Additionally, we revealed the effect of soyasapogenol on the differentiation of adipocytes associated with the expression levels of specific transcription factors, PPARγ and FAS. However, no significant expression levels were observed in the three groups on decreasing triglyceride accumulation in adipocytes. Further studies are warranted to determine whether soyasapogenol affects the expression of other specific transcription factors.

Finally, we measured the concentrations of secreted adiponectin and resistin, known as the representative adipocytokines involved in diabetes. These adipocytokines that are reported to be secreted from 3T3-L1 cells have different functions [27], [28]. Adiponectin reduces glucose and lipid levels by inhibiting hepatic gluconeogenesis and lipogenesis [29], [30]. Conversely, resistin inhibits insulin signals, inducing insulin resistance [28]. Resistin is also reported to be involved in inflammation [31]. As shown in Fig. 5A, the secreted adiponectin levels in adipocytes treated with soyasapogenol A were significantly higher than those treated with the vehicle during days 6–10. In a similar way with soyasapogenol A, adiponectin levels in adipocytes treated with soyasapogenol B were significantly higher during days 4–10. However, there were no significant differences in adiponectin levels between the cells treated with soyasapogenol A or soyasapogenol B. Adiponectin secretion levels are known to be higher in small adipocytes with a lower lipid accumulation [32]. The current results indicated that soyasapogenol A and B might affect lipolysis by increasing the number of small adipocytes, followed by an enhancement of adiponectin secretion.

By contrast, resistin is known to be abundantly secreted from large adipocytes that store a much higher lipid content [33]. Our results clearly indicate that resistin secretion in soyasapogenol-treated adipocytes was significantly decreased compared with that in vehicle-treated adipocytes during days 6–10 (Fig. 5B). These findings suggest that the lowered cellular triglyceride levels in soyasapogenol-treated adipocytes were related to the reduction in resistin secretion. Therefore, an increase in adiponectin secretion and decrease in resistin secretion are considered to have a synergistically beneficial effect on inhibiting the progress of MS associated with obesity and diabetes.

Intriguingly, the potency of anti-obesity and enhancement of lipolysis were observed to be different between soyasapogenol A and soyasapogenol B. Soyasapogenol B was more potent than soyasapogenol A on anti-obesity-related effects in the present study. As shown in Fig. 1, the structure of soyasapogenol A and B is very similar, but they differ in their number of hydroxyl groups. The structure-dependent differences of physiological functions might lead us to speculate that molecular targets of soyasapogenol such as transcription factors or G protein-coupled receptors exist. Further studies will be needed to clarify why the potency on anti-obesity-related effects is different between soyasapogenol A and B.

Recently, we found that the anti-obesity effect of soyasapogenol B was shown in mice fed a high-fat diet [34]. This observation might be consistent with our present results using cultured adipocyte cells. These lines of evidence indicate that soyasapogenol, especially soyasapogenol B, might be a useful anti-obesity therapeutic agent as a dietary food or supplement.
