# Supplementary material for: Soyasapogenols reduce cellular triglyceride levels in 3T3-L1 mouse adipocyte cells by accelerating triglyceride lipolysis
Source: Biochem Biophys Rep. 2018 Sep 27;16:44–9. doi: 10.1016/j.bbrep.2018.09.006 (PMC6171538; doi:10.1016/j.bbrep.2018.09.006)
Supplement: Supplementary file 1 — Supplementary material [file mmc1.docx]

There is no Conflict of Interest in our manuscript.
